# Progression from Beryllium Exposure to Chronic Beryllium Disease: An Analytic Model

**DOI:** 10.1289/ehp.0800440

**Published:** 2009-02-27

**Authors:** Philip Harber, Siddharth Bansal, John Balmes

**Affiliations:** 1 Division of Occupational and Environmental Medicine, Department of Family Medicine, University of California at Los Angeles, Los Angeles, California, USA; 2 Division of Occupational and Environmental Medicine, Department of Medicine, University of California at San Francisco, San Francisco, California, USA

**Keywords:** beryllium, beryllium sensitization, chronic beryllium disease, cost-effectiveness, screening

## Abstract

**Background:**

Understanding the progression from beryllium exposure (BeE) to chronic beryllium disease (CBD) is essential for optimizing screening and early intervention to prevent CBD.

**Methods:**

We developed an analytic Markov model of progression to CBD that assigns annual probabilities for progression through three states: from BeE to beryllium sensitization and then to CBD. We used calculations of the number in each state over time to assess which of several alternative progression models are most consistent with the limited available empirical data on prevalence and incidence. We estimated cost-effectiveness of screening considering both incremental (cost/case) and cumulative program costs.

**Results:**

No combination of parameters for a simple model in which risk of progression remains constant over time can meet the empirical constraints of relatively frequent early cases and continuing development of new cases with long latencies. Modeling shows that the risk of progression is initially high and then declines over time. Also, it is likely that there are at least two populations that differ significantly in risk. The cost-effectiveness of repetitive screening declines over time, although new cases will still be found with long latencies. However, screening will be particularly cost-effective when applied to persons with long latencies who have not been previously screened.

**Conclusions:**

To optimize use of resources, the intensity of screening should decrease over time. Estimation of lifetime cumulative CBD risk should consider the declining risk of progression over time.

Chronic beryllium disease (CBD) is an increasingly recognized occupational health problem (Newman et al. 2001, 2005). Three categories of health status with respect to CBD have been identified: *a*) beryllium exposed without sensitization (BeE), *b*) beryllium-sensitized without disease (BeS)—presence of blood lymphocytes with *in vitro* proliferation in response to beryllium, and *c*) CBD—a chronic granulomatous disease that involves a beryllium-specific cell-mediated immune response and that is similar in clinical presentation to sarcoidosis (Rossman 1996; Williams 1996). CBD predominantly affects the lungs and may lead to severe disability or death (Rossman 2008). Currently, a two-stage screening process is used. The first stage of screening for this immunologic disorder (Rossman 1996; Saltini et al. 1998) is applied to exposed individuals and is based upon testing for lymphocyte proliferation to beryllium stimulation. Those with positive results undergo detailed clinical assessment with more extensive testing such as pulmonary function testing, high-resolution computed tomography scans, and fiber optic bronchoscopy with transbronchial biopsy (Maier 2002; Rossman 1996).

The large population of workers and community members (Maier et al. 2008) with potential exposure makes it important to understand the frequency and time course of development of both sensitization and CBD among exposed individuals. The available clinical and epidemiologic data are not adequate to fully characterize the processes of sensitization and development of lung disease. Although the literature concerning treatment is limited (Marchand-Adam et al. 2008; Preuss 1985; Rossman 2008; Sood et al. 2004), it appears likely that there is benefit of screening and early treatment in many cases. Therefore, we have developed an analytic approach to model progression from BeE to BeS and from BeS to CBD with the goal of optimizing screening among exposed populations (Table 1).

## Methods

We used the following stepwise approach: review of relevant published research studies and case series to delineate model constraints based upon empiric data, development of a series of possible analytic models to describe the progression from exposure through sensitization to CBD, evaluation of models based on compatibility with the constraints imposed by the empiric data, and use of model-generated information to assess cost-effectiveness of screening.

**Figure 1:**
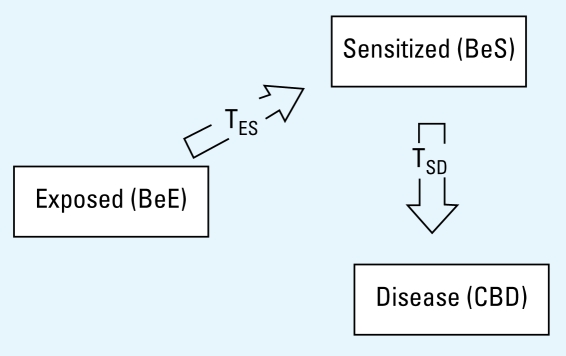
Three-state progression model and annual transition probabilities T_ES_ and T_SD_.

A Markov analytic model was employed (Goldie 2003; Sonnenberg and Beck 1993) and a series of health states and transition probabilities that express the likelihood of moving to another state in any time period (e.g., from healthy to early disease) are defined. The model applied annual transition probabilities for progression from BeE to BeS and from BeS to CBD. We assumed that transitions are unidirectional and that CBD is an absorbing state (i.e., there is no transition out of the state). Calculations were performed using commercially available software (TreeAge Pro Suite 2007, release 1.2; TreeAge Software Inc., Williamstown, MA; Microsoft Excel). Figure 1 shows the basic model, which was built considering a sample population of 1,000 BeE individuals who were BeS and CBD free at the starting point, and assigning an annual transition probability to each of the two possible transitions, from BeE to BeS (T_ES_) and from BeS to CBD (T_SD_).

For each year over a 20-year span, the transition probabilities were applied to the individuals in each state at the beginning of the year to calculate the number progressing to a new state during the year. The number of BeE individuals was multiplied by T_ES_ to determine the number who advanced to BeS, and the number in the BeS state was multiplied by T_SD_ to determine how many developed CBD. We generated graphical displays to show changes in the distribution among the three states over time.

Various combinations of T_ES_ and T_SD_ parameters were evaluated to compare distributions and evaluate consistency with available empirical data. We then enhanced the basic model first by using time-dependent transition probabilities to evaluate the effect of latency on disease progression. Two latency-effect parameters were permitted for each annual proportionate increase or decrease in the transition probability and the year at which the change began (e.g., a 1% annual decline in T_ES_ starting with the fifth year of latency). We then used mixed population models to evaluate the assumption that two distinct populations that differ in risk (i.e., different T_ES_ and T_SD_ parameters) are present among BeE individuals. This is a reasonable assumption given the observed difference in risk between individuals with or without a glutamic acid residue in the 69th position of the β-chain of the human leukocyte antigen (HLA) DP allele (Glu^69^). The overall observable distributions are determined by combining results, weighted by the relative proportions. We included both time-dependent transition probabilities and the mixed population assumption in a more complex model.

Cost-effectiveness was evaluated by considering the yield of new cases detected in relationship to the cost of screening. Screening for beryllium-related health effects includes two components: annual blood lymphocyte proliferation testing for persons with BeE to detect BeS, and in-depth evaluation every 3 years (triennially) with pulmonary function testing, chest imaging, and possible bronchoscopy to detect CBD among persons with BeS. For each year, we calculated the number of new BeS and CBD cases as the difference between those present before the year and at the end of the year. Cost was estimated by applying a standardized cost to each blood test performed and in-depth evaluation performed (€100 and €5,000, respectively). Cost estimates were converted to Euros to prevent misunderstanding that they were directly observed from empirical U.S. data. Both annual and cumulative program costs to date were calculated.

Several metrics expressed relationships between yield and cost: incremental cost per new case detected is equal to the total program cost for a year divided by the number of CBD cases detected in that year; cumulative average cost per case to date is equal to the total cumulative program costs to date divided by total number of cases to date; and case yield is equal to the proportion of assessments that are positive, calculated for the first and second stages of screening as BeS/BeE or CBD/BeS, respectively.

## Results

### Selection of parameters from empirical data

From several cross-sectional surveys, we determined a reasonable range of prevalence for BeS and CBD (Table 1) (Cummings et al. 2007; Donovan et al. 2007; Henneberger et al. 2001; Kreiss et al. 1989, 1996, 1997; Newman et al. 2001; Rosenman et al. 2005; Sackett et al. 2004; Stange et al. 2001; Welch et al. 2004). The range of BeS extends from < 1% (Sackett et al. 2004) to 12% (Kreiss et al. 1989). CBD prevalence ranges from 0.1% to 9.1% (Henneberger et al. 2001; Welch et al. 2004). Studies show a greater BeS prevalence with longer latencies (Cummings et al. 2007; Stange et al. 1996; Viet et al. 2000) and a consistent increase of prevalence with increasing latency (i.e., a monotonic effect) (Henneberger et al. 2001). However, both BeS and CBD also develop with short latency (e.g., 4 of 15 cases with less than 3 months of exposure; Newman et al. 2001). A few studies (Newman et al. 2001; Stange et al. 1996) have reported periodic rescreening, allowing determination of apparent incidence. Such studies are usually relatively small and over a short time course of 2–3 years. Therefore, we did not directly apply the reported incidence rates for comparing models.

Based on the data from the reviewed studies, several constraints for assessing models were adopted: Both CBD and BeS can develop with short latencies; new cases of both BeS and CBD continue to develop after many years of latency, and even with very long latencies; and most exposed workers develop neither BeS nor CBD.

### Models

We employed several models, ranging from simple to more complex models; Table 2 summarizes several examples. In addition to the specific parameter estimates shown, other combinations of parameter estimates were evaluated.

### Simple model (fixed transition probabilities)

**Figure 2:**
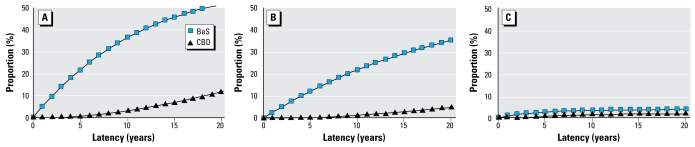
Progression over time: proportion of persons in BeS and CBD states for each year. (*A*) Single population, constant transition probabilities (model A). (*B*) Mixed population, constant transition probabilities (model D). (*C*) Mixed population, time-dependent probabilities (model E). For model definitions, see Table 2.

As shown in Figure 2, A and B, applying unchanging transition probabilities leads to relatively few cases in the early years. Additionally, the proportion with CBD among those with positive blood lymphocyte proliferation tests for BeS is quite low in the early years of screening. Use of transition probabilities that yield adequate prevalence of BeS and CBD with short latencies leads to excessive prevalence of both BeS and CBD in latter years. Therefore, it is quite unlikely that the simple model, with constant annual transition probabilities, is accurate.

### Incorporation of time-dependent latency factors

Incorporation of a negative latency factor (decline in risk of progression over time since first exposure) helps meet the constraint of relatively high prevalence in the early years without proportional increases in later years. Furthermore, if the decline in risk of progression over time is greater for BeS than for CBD development, the CBD:BeS ratio does not increase too markedly over time. However, the predicted prevalence does not approximate those reported in empiric studies. Parameters that yield sufficiently high incidence rates lead to inappropriately high prevalence after the first few years. Therefore, it appears unlikely that this model adequately represents the course of progression.

### More complex models

The model that provided the best fit with the empirically derived constraints incorporated both time-dependent transition probabilities and a mixed population assumption (Figure 2C).

### Screening cost-effectiveness

**Figure 3:**
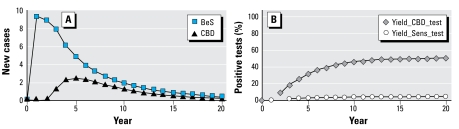
Incidence by year. (*A*) Number of projected new cases for each year. Results are based on model E (mixed population, with latency time-dependent transition probabilities; see Table 2). (*B*) Percentage of tests (“yield”) that will be positive if the test is applied in the specified year. “Yield_CBD_Test” refers to the proportion of positive detailed tests for CBD among persons with BeS, and “Yield_Sens_Test” refers to the proportion of positive tests for BeS among those in the BeE state.

**Figure 4:**
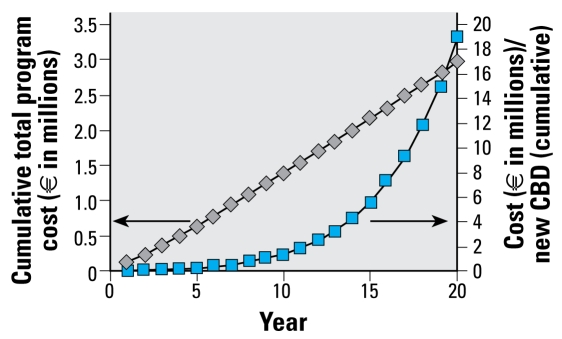
Estimated cost-effectiveness of screening using the assumptions for model E (time-dependent progression probabilities in a mixed population; Table 2).

For cost-effectiveness analyses, we used the model incorporating time-dependent transition probabilities and a mixed population assumption. Figure 3 shows the annual incidence of BeS and CBD and illustrates the percentage of positive tests for BeS and CBD. Table 3 and Figure 4 show results for periodic screening with annual blood testing of BeE individuals and triennial in-depth evaluations for CBD of BeS individuals. The figures show that the annual number of new cases increases for the first few years and then declines. In addition, the cost-effectiveness of repetitive screening declines over time. When the screening program is applied to a population over many years, both the incremental cost of finding a new case and the average cumulative cost per case increase with latency.

Table 3 shows the impact of screening applied to previously untested populations. Results are shown for incremental and cumulative costs for new cases of CBD and BeS with periodic screening. In addition, the right most column of the table shows the cost per case of CBD if screening is applied only one time; the table shows the cost per case according to the latency time at which the one-time screening is performed. Cost-effectiveness is greater in this situation because it detects prevalent rather than incident cases. Furthermore, the cost per case is relatively low even when screening is implemented for workers with long latencies since onset of exposure.

## Discussion

To optimize screening and early intervention programs to prevent progression to severe disease, several questions must be answered: *a*) How rapidly do individuals with exposure develop BeS? *b*) How likely are BeS individuals to develop CBD? *c*) What is the time course of these changes? *d*) Does the risk change over time since initial exposure? *e*) How cost-effective are screening methods for BeS and CBD?

Empirical studies of occupational cohorts (Cummings et al. 2007; Henneberger et al. 2001; Newman et al. 2001; Stange et al. 1996; Viet et al. 2000; Yoshida et al. 1997) and reports of clinical series are inadequate for fully describing the course of progression (Harris et al. 1997; Kreiss et al. 1993; Maier et al. 2002; O’Brien et al. 1987; Preuss 1985; Rees 1979). However, the available data permit constraints on possible ranges of the parameters of disease progression to be defined. Although differing opinions about the value of screening for BeS and disease have been presented (Borak et al. 2006; Cullen 2005; Rossman 2008), there is evidence that both BeS and CBD can be detected in early stages and that treatment with corticosteroids or other medications can be beneficial (Rossman 2008; Sood et al. 2004). The present analysis may help inform the debates about the utility of screening; for example, it adds information about the likelihood and time course of progression from BeS to CBD. Differences in the reported prevalence of BeS and CBD among studies are possibly due to exposure level differences, mis-classification of exposure status, or dissimilar follow-up. Because residents of communities near beryllium production facilities are also at risk of developing CBD and BeS (Maier et al. 2008), similar analyses may be appropriate for informing screening programs for large community populations with relatively low exposure (Redlich and Welch 2008).

We applied a Markov simulation model to assess possible assumptions about the risk of progression. Available empirical information includes cross-sectional prevalence of BeS and CBD soon and many years after initial exposure, and the relationship between numbers of individuals with BeS and CBD. We based our basic model upon the three widely accepted states of beryllium-related health status (BeE, BeS, CBD). Although there is residual uncertainty in the precise values of the two annual transition probabilities (BeE to BeS and BeS to CBD, respectively), the patterns of the distributions under different assumptions are sufficiently different to allow meaningful contrasts. Thus, the time-varying transition-probability mixed-population model was most appropriate across the range of prevalence studies in the published literature.

It is unlikely that the annual risk of development of BeS and/or CBD remains constant. A simple model with constant annual rates of progression cannot yield prevalence estimates consistent with relatively high prevalence within the first 5 years of exposure (Henneberger et al. 2001; Newman et al. 2001) and continued development of new cases of BeS and CBD many years after initial exposure (Cummings et al. 2007; Henderson 1970; Henneberger et al. 2001; Newman et al. 2001; Stange et al. 1996). Rather, this risk is likely to decline with increasing latency.

Inclusion of two populations differing in risk of progression and their respective declines in risk over time improves the fit with the empirical data. Such assumptions are biologically and epidemiologically reasonable. CBD is one of the best examples of gene–environment interaction. Several genes, particularly the Glu^69^ variant in the β-chain of the HLA-DP allele, are strongly associated with individual risk, making it biologically likely that there are at least two groups in the exposed population groups that have different susceptibility toward progression. Furthermore, job title is closely associated with risk, so machinists have considerably greater risk than do lesser exposed workers (Newman et al. 2005). Temporal decline in annual risk would occur as the higher risk persons develop BeS and CBD, thereby reducing the average risk of those who remain at risk of progression.

### Optimizing screening programs

The declining annual risks of developing new BeS or CBD suggest that screening intensity may be reduced as time since initial exposure increases. This would allow effective focusing of available resources. For example, the frequency of repeated blood lymphocyte proliferation testing of persons with prior exposures should be greater in the early years rather than in later years. Calculations may understate the impact because our models did not incorporate measures of health benefit or health risks. Because persons with long latencies tend to be older, years of life or quality adjusted years of life saved would be lower in long-latency cases. Similarly, the health risks of diagnostic procedures (e.g., bronchoscopy) and of treatment (e.g., high-dose prednisone) are likely to be greater in long-latency cases.

Most of the empirical studies generally do not clearly distinguish years since first exposure from years since last exposure. Therefore, these results should not be interpreted to suggest reduced screening intensity of currently exposed workers who have long latency. However, a high proportion of individuals being screened ceased being exposed many years ago; for example, many had been employed in the former nuclear weapons industry.

Nor do these results apply to persons who present with relevant clinical evidence suggestive of CBD such as radiographic signs (e.g., interstitial or ground glass opacities), pulmonary function abnormalities (e.g., reduced diffusing capacity), or incidental findings on biopsy (e.g., granuloma or lymphocytic infiltrate). Indeed, the reduction over time of screening cost-effectiveness when applied nonselectively argues for focusing resources upon those with higher likelihood of remediable disease.

The results of our more complex models also support the benefit of screening individuals or populations that have not been previously tested. Table 3 shows that even with long latency, both cost-effectiveness and diagnostic yield are significant when applied to exposed populations not previously tested. Under such circumstances, the screening seeks to identify prevalent rather than incident cases. Therefore, there will be a pool of cases that have accumulated over many years.

### Limitations

Our models do not provide precise estimates of incidence rates and prevalence over time. Nevertheless, they demonstrate that risk of progression declines with time and provide useful insights into optimization of screening programs.

There are significant data gaps in the available population and clinical studies. These studies report divergent prevalence values for several possible reasons. Case definitions for both BeS and CBD differ among studies. The populations are heterogeneous in terms of length and magnitude of exposure. This affects the prevalence because the risk of BeS and CBD is dose related (Henneberger et al. 2001; Viet et al. 2000; Yoshida et al. 1997). Prevalence is also affected by inclusion of retirees (Cummings et al. 2007; Stange et al. 1996). The study populations are heterogeneous. Cross-sectional studies include individuals with both short and long latencies. The cross-sectional studies are subject to survivor and ascertainment bias; those who had severe CBD and those who have left the worksite would not appear in several of the studies. Adequate, long-term cohort studies are absent.

We simplified the calculations by using a 1-year “time slice” for applying transition probabilities. A person developing BeS at the beginning of a year would not be considered part of the pool at risk of progressing to CBD until the end of the year. Similar considerations apply to calculating diagnostic yield for triennial in-depth evaluation based upon the average size of the BeS population over that time. Such errors are likely to be relatively small.

The cost data are somewhat arbitrary, and the cumulative cost models do not incorporate either cost inflation or discounting of later versus early expenditures. However, the modeling effectively demonstrates the relative changes in cost-effectiveness. Similar approaches have been applied to occupational asthma (Wild et al. 2005) and for selecting workers for spirometry screening (Schwartz et al. 1988). The analysis includes only the direct cost of the testing (e.g., cost per subject tested) and did not include fixed program costs (e.g., program administration) or indirect costs (e.g., lost work time during testing). Furthermore, the approach treated screening with blood beryllium lymphocyte proliferation tests as a single entity; alternative algorithms of test and rapid retest have been suggested (Middleton et al. 2006).

In summary, combining published observational data and several possible progression models suggests that the risk of developing BeS is greatest in the first few years after exposure and then declines, and that the annual risk of progressing from BeS to CBD declines over time. However, there is a persistent risk of developing new BeS and new CBD even with long latency, so screening intensity should be adjusted according to years of latency in order to optimally use resources. Screening is also useful for exposed workers who have not been previously tested.

## Correction

In the original manuscript published online, the standardized costs applied to each blood test and in-depth evaluation were incorrect. They have been corrected here.

## Figures and Tables

**Table 1:** Examples of studies reporting prevalence.

Reference	No.	BeS + CBD prevalence (%)	Latency	CBD prevalence (%)	Latency
Donovan et al. 2007	277	10.50	S		
	539	9.10	L		
Henneberger et al. 2001	151	9.50	S	1.40	S
		10.40	L	9.10	L
Kreiss et al. 1989	51	11.70	L	7.80	
Kreiss et al. 1996	136	3.70	S	3.70	S
Kreiss et al. 1997	627	6.90	L	4.60	L
Newman et al. 2001	235	6.40	L	3.80	
Rosenman et al. 2005	577	7.00	L	7.60	L
Sackett et al. 2004	2,221	0.80	L	0.14	
Schuler et al. 2005	153	6.50	L	3.92	L
Stange et al. 1996	4,268	1.70	L	0.60	L
Welch et al. 2004	3,842	1.40	L	0.10	L

Abbreviations: L, long (e.g., 10–20 years); S, short (e.g., < 5 years). “BeS + CBD” includes all subjects with positive blood beryllium lymphocyte proliferation tests.

**Table 2:** Models employed.

		Transition probability^a^	
Model	Population type	T_ES_	T_SD_
A	Single	Constant	Constant
		5%	2%

B	Single	Time dependent	Constant
		10%, then decreasing by 20% each year starting at year 4	5%

C	Single	Time dependent	Time dependent
		10%, then decreasing by 20% each year starting at year 4	50%, then decreasing by 35% each year starting at year 3

D	Mixed^b^	Constant	Constant
	Glu^69^ positive	5%	2%
	Glu^69^ negative	2%	1%

E	Mixed	Time dependent	Time dependent
	Glu^69^ positive	2.5%, then decreasing by 20% each year starting at year 4	20%, then decreasing by 20% each year starting at year 3
	Glu^69^ negative	0.25%, then decreasing by 10% each year starting at year 4	2%, then decreasing by 10% each year starting at year 3

a Transition probabilities for annual risk of progressing from BeE to BeS (T_ES_) and from BeS to CBD (T_SD_) for each model

b Glu^69^ positive and negative refer to variants in the β-chain of the HLA-DP allele.

**Table 3:** Cost-effectiveness for periodic screening: annual blood testing for BeE individuals and triennial in-depth evaluations for CBD for BeS individuals.

Year (latency)	New cases		Incremental cost (€)/new case		Cumulative cost (€)/CBD case	Cost (€)/CBD one-time screen
	BeS	CBD	BeS	CBD		
1	9.4	0.0	10,538	0		
2	9.0	1.3	10,970	57,482	184,387	135,152
3	8.0	2.3	12,226	44,301	161,214	56,300
4	6.1	2.5	15,794	48,905	205,505	36,492
5	4.9	2.3	19,861	58,655	282,278	28,136
6	3.9	2.0	24,538	72,212	393,245	23,710
7	3.2	1.7	29,938	89,716	546,571	21,054
8	2.6	1.4	36,183	111,747	754,022	19,331
9	2.2	1.2	43,403	139,129	1,030,902	18,155
10	1.8	1.0	51,736	172,898	1,396,560	17,323
11	1.5	0.8	61,331	214,296	1,875,121	16,720
12	1.3	0.7	72,350	264,790	2,496,376	16,273
13	1.1	0.6	84,966	326,092	3,296,782	15,939
14	1.0	0.5	99,370	400,176	4,320,608	15,686
15	0.8	0.4	115,765	489,307	5,621,186	15,493
16	0.7	0.3	134,374	596,054	7,262,187	15,345
17	0.6	0.3	155,441	723,319	9,319,138	15,232
18	0.5	0.2	179,230	874,355	11,880,908	15,144
19	0.5	0.2	206,033	1,052,775	15,051,192	15,077
20	0.3	0.2	353,315	1,262,579	18,950,309	15,025

Data include the number of new cases each year for a population of 1,000 BeS persons initially free of abnormality. Cumulative cost/case is the cost to date/total cases to date. Calculations are based on model E (Table 2).
